# Utility of Cupping Therapy in Substance Use Disorder: A Novel Approach or a Bizarre Treatment?

**DOI:** 10.7759/cureus.47445

**Published:** 2023-10-21

**Authors:** Viren Solanki, Shashwat Mallik, Shahin Khan, Vedant Desai, Joseph Pergolizzi

**Affiliations:** 1 Psychiatry, Gujarat Medical Education and Research Society (GMERS) Medical College and Hospital, Valsad, IND; 2 Medicine, Government Medical College, Baroda, Vadodara, IND; 3 Medicine, Gujarat Medical Education and Research Society (GMERS) Medical College and Hospital, Valsad, IND; 4 Anesthesiology and Pain Medicine, NEMA Research, Inc., Naples, USA

**Keywords:** complementary and alternative medicine (cam), nicotine use disorder, opioid use disorder (oud), benzodiazepine dependence, substance use disorders, alternative medicine, de-addiction, cupping therapy

## Abstract

Substance use disorder is a psychiatric problem not bound by age, sex, ethnicity, sexual preference, geography, socio-economic status, educational level, or political and religious ideologies. While robust pharmacotherapy and psychotherapy treatments are available for de-addiction and managing withdrawal symptoms, patients from rural areas and lower socio-economic classes often prefer alternative medicine. Cupping therapy is one such ancient practice used mainly for organic physical conditions. A patient addicted to alprazolam, codeine, and tobacco presented to our psychiatry outpatient department for de-addiction and management of his withdrawal symptoms. He came to seek professional help after a trial of cupping therapy by an alternative medicine practitioner, which did not improve his condition. His withdrawal symptoms subsided after standard treatment. As found in this case, cupping therapy is not beneficial in treating substance use disorder or managing withdrawal symptoms. Awareness of the utility and consequences of cupping therapy and other alternative therapies is required to promote rational scientific treatments. Substantial reforms in health promotion and health education are required to educate the general population regarding the most effective treatments available, and the risks of iatrogenesis associated with traditional cures that are not evidentially backed.

## Introduction

Substance use disorder is a major psychiatric public health problem that plagues every stratum of society and impacts all spheres of life regardless of age, sex, ethnicity, sexual preference, geographical region, financial or social status, literacy, or political and religious ideologies [1]. While legislative efforts have made counseling, treatment, and rehabilitation accessible to most of the population, the public's belief in alternative medicine and taboos regarding mental health have been massive roadblocks in promoting safe and scientific mental health services. Patients from rural backgrounds are more likely to prefer alternative forms of medicine or unqualified professionals practicing faith healing or rituals as opposed to seeking standard mental health care [2,3].

Cupping therapy is an ancient therapy that has claimed to treat numerous organic physical conditions, albeit without scientific evidence. While there are some reports suggesting its efficacy in different physical health conditions, its utility in mental health diseases is largely unknown, other than a couple of isolated studies [4-7]. Numerous traditional treatments have been explored for mental health diseases, most without any scientific backing, and there needs to be more caution in this aspect. Other than the lack of effectiveness of many such procedures, some may also predispose individuals to complications, also known as iatrogenesis [8,9]. A greater focus needs to be placed on the evidence-based pharmacotherapy and psychotherapy of these psychiatric illnesses, including substance abuse [10-13]. Here, we are reporting a case of a patient with substance use disorder who underwent cupping therapy before presenting to the psychiatry outpatient department of our tertiary care hospital.

## Case presentation

**Figure 1:**
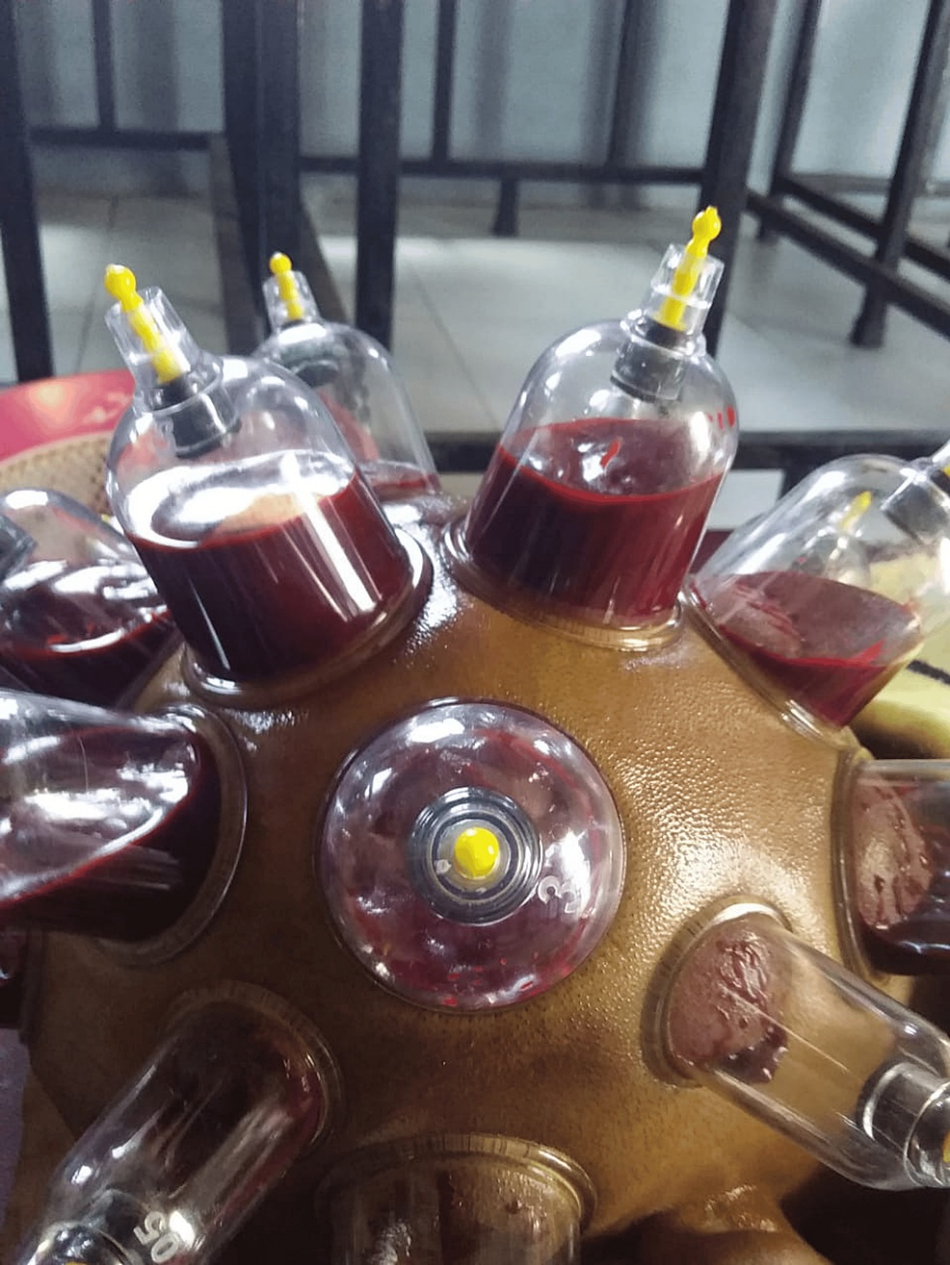
Wet cupping therapy on the head with blood oozing from incision sites

A 30-year-old man presented to the psychiatric outpatient department with complaints of nervousness, anxiety, a sense of impending doom, irritability, anger outbursts, headache, and reduced sleep and appetite for the last five days. The patient had a history of daily consumption of 5-10 mg of alprazolam tablets, 200-250 mg of codeine syrup, and about five packets of chewable tobacco over the last seven years; this was a pattern of polyaddiction to a benzodiazepine, opiate, and nicotine. The patient had no history of fever, confusion, or hallucinations. On eliciting the past history, the patient revealed that he went to an alternative medicine practitioner after his family persuaded him to seek help for his substance use disorder. After ceasing the consumption of all three substances for three days, he started developing the symptoms with which he presented to our hospital. He was hesitant to talk about his substance use disorder to medical professionals and concerned about confidentiality, and, hence, went to an alternative medicine practitioner whom he deemed approachable. There he was given wet cupping therapy on the head for four days, which involved the use of rubber pumps to create a suction inside the cups placed on his head. After three to five minutes, the cups were removed and small incisions were made on the cupping sites, following which a second suction caused the oozing out of blood from the incision sites on the scalp (Figure 1). But, this did not improve his symptoms, and hence, he stopped going there two days before coming to our tertiary care hospital.

**Figure 2:**
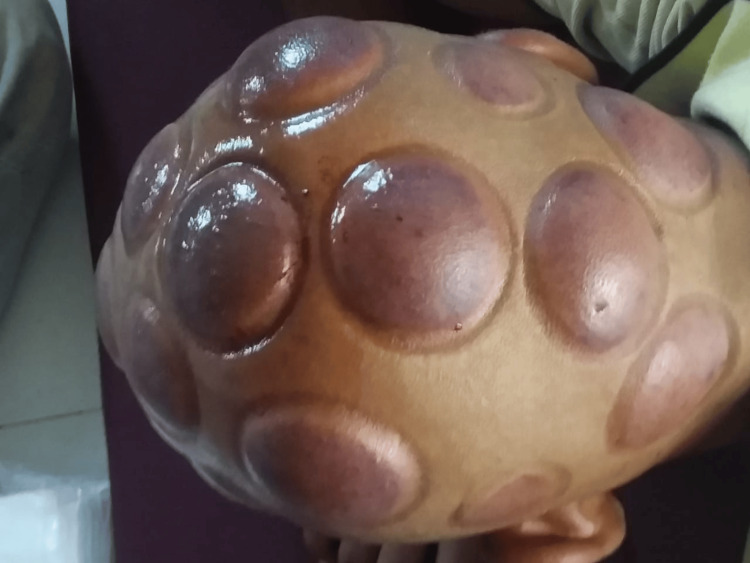
Cupping marks on the head

On examination, the patient had a pulse rate of 76 beats per minute, blood pressure of 128/78 mm Hg, and respiratory rate of 22 per minute. He was well-oriented to time, place, and person. Systemic examination of the cardiovascular system was unremarkable. He denied any other substance use. The skin over his head had distinct cupping marks but no signs of infection or active bleeding, which are some common complications after cupping therapy (Figure 2). On assessment, the patient had a Clinical Opiate Withdrawal Scale (COWS) score of 13 and a Clinical Institute Withdrawal Assessment (CIWA) scale score of 26.

Later, the patient was admitted to the psychiatric ward to manage the withdrawal symptoms, where we initiated pharmacotherapy. Tablet diazepam (20 mg/day), sodium valproate (800 mg/day), tramadol (200 mg/day), thiamine (300 mg/day), paracetamol (500 mg/day) and intravenous fluids were given to the patient. We counseled the patient regarding substance abuse, its harmful effects, and de-addiction. The patient's symptoms started to improve, and we continued the treatment for four days and discharged him with a COWS score of 4 and a CIWA score of 2. We intended to reassess him after 14 days, but we lost him to follow-up.

## Discussion

Addiction has emerged as a threat to humankind, plaguing various ages ranging from childhood to old age. It not only includes substance addiction but also non-substance addiction. Instead of a single etiology, addiction is multifactorial with a complex association of environmental, genetic, and personal responses. Multiple variables dependent on the individual, the substance, and the environment along with a genetic predisposition are responsible for the development of this complex disease [14]. Over the years, the biological basis of substance use disorder has gained the limelight. Addictive substances affect the reward pathways of the brain, which positively reinforce their use and also stimulate areas (dorsal striatum of the basal ganglia) involved in habit formation. Along with social, behavioral, genetic, environmental, and substance-related components, it is also a chronic brain disease due to the effects of drug abuse [15].

Substance use disorders form a large chunk of addiction disorders, which cause distress to the patients as well as those around them. Modern medicine and psychiatry offer scientifically proven and safe de-addiction options consisting of both pharmacotherapy and psychotherapy, as well as the treatment of withdrawal symptoms [10-13]. Our patient presented with a polyaddiction of benzodiazepines, opiates, and nicotine. Benzodiazepine dependence is common with short-acting drugs like alprazolam. The treatment options include withdrawal with abstinence, benzodiazepine maintenance therapy, psychotherapy, and substitution therapy with longer acting alternatives such as diazepam. Flumazenil and anti-epileptics are some other pharmacological alternatives that may be helpful as seizures have even been recorded two to three weeks post-abstinence [10]. Codeine is one of the most accessible opiates for abuse, and tramadol, methadone, and buprenorphine are the common pharmacological agents used to treat opiate use disorders. Opiate use disorder is extremely dangerous owing to its strong depressant action, which can lead to cardiac arrest and respiratory arrest [11]. Nicotine, the most addictive and harmful substance in smoking and tobacco chewing, is one of the most commonly abused drugs globally, and while behavioral therapies exist, chronic smokers or tobacco chewers may find it difficult to overcome it. Hence, pharmacological modalities like nicotine replacement therapy have gained more importance in the past few decades. Non-nicotinic agents like varenicline and bupropion have also proved beneficial in the de-addiction of tobacco chewers [12].

But in rural and remote areas riddled with taboos towards mental health discussions and over-reliance on alternative medicine, mental health professionals are often only the second point of contact for patients who are unaware of the evidential superiority that modern medicine possesses. As seen in this case, our patient preferred to visit an unqualified individual due to fear of stigmatization if he were to visit a psychiatrist. This can be attributed mainly to the social norms and cultural taboos prevalent in rural parts of India, as government health services do not partake in the spread of misinformation. However, a more proactive approach to advertise the benefits of evidence-based medicine should be undertaken at the grassroots level.

Cupping therapy is an ancient practice claiming to help those with blood disorders, rheumatological diseases, gynecological diseases, dermatological problems, vascular disorders, and allergic conditions. However, the scientific evidence for these is sparse [13,16]. There have been multiple proposed mechanisms of action, including immunomodulation theory and genetic theory [17]. As seen in our patient, cupping therapy has no utility in treating polyaddiction or the associated withdrawal symptoms. This is in contrast to some studies that suggest it is efficacious in the treatment of depression and the cessation of smoking [5,6]. The practice is not entirely harmless but carries a risk of minor burns, bruises, and skin infections. Wet cupping involves making small cuts in the skin, followed by applying suction to draw out small amounts of blood. This has an additional peril of transmission of blood-borne diseases if the sterility of the equipment is not maintained [7]. Although not seen in this case, cupping therapy could be a reason for iatrogenesis in such patients if its unscientific usage in substance addiction continues. Acupuncture, traditional herbal medicines, and mind-body interventions are other popular alternative therapies for substance use disorders. These alternate therapies require extensive scrutiny with large-scale human trials before advertising them to the patients, to prevent cases of medical iatrogenesis [9,18-20].

## Conclusions

Established treatment modalities for substance use disorder and its withdrawal symptoms include pharmacotherapy and psychotherapy, but their utilization by the general population remains unsatisfactory. Taboos regarding mental health services and concerns about confidentiality are massive obstacles for patients seeking psychiatric help, and alternative forms of medicine may seem more approachable, even with the associated risks. As displayed in this case, cupping therapy is a traditional therapy with no role in treating polyaddiction and withdrawal symptoms, but it unnecessarily exposes individuals to really uncomfortable and often concealed complications such as bruising, and skin and blood infections, especially when carried out by untrained, incompetent individuals. While one can explore these options in addition to seeking professional mental health care, it is imperative to spread awareness about the roles, scientific soundness, and adverse effects of these alternative health practices. The health promotion and education sectors need reforms to educate the general population, especially the rural population in India, about the dangers of iatrogenesis caused by non-evidence-backed treatments. There needs to be an extensive advertisement of only the most effective and scientific treatment options provided by medical professionals, and the risks of overlooking them in favor of traditional cures propagated by unqualified individuals. With all the scientific advancements in the 21st century ranging from artificial intelligence in healthcare, and robotic surgeries, to extensive clinical trials for novel anti-cancer drugs, we cannot allow the propagation of ancient, scientifically unsound techniques that may cause more harm than benefit to patients.

## References

[1] Daley DC (2013). Family and social aspects of substance use disorders and treatment. J Food Drug Anal.

[2] Adams J, Sibbritt D, Lui CW (2011). The urban-rural divide in complementary and alternative medicine use: a longitudinal study of 10,638 women. BMC Complement Altern Med.

[3] Ray J, Chakrabarty D, Paul R, Som K (2018). Prevalence of the use of complementary and alternative medicine in an eastern Indian population with emphasis on tribal/ethnic minority groups. J Taibah Univ Med Sci.

[4] Cao H, Han M, Li X (2010). Clinical research evidence of cupping therapy in China: a systematic literature review. BMC Complement Altern Med.

[5] Noor S, Haider S, Fatima F, Mumtaz M (2021). Al-Hijama - a possible cure for depression: a pilot study. Sch Int J Tradit Complement Med.

[6] Saeed AM, Mohammed RM, Aty Ibrahim MEA (2015). Evaluation of cupping therapy as an adjuvant therapy in a smoking cessation program. Egypt J Bronchol.

[7] (2022). What is cupping therapy? Uses, benefits, side effects, and more. https://www.webmd.com/balance/cupping-therapy.

[8] Yeung KS, Hernandez M, Mao JJ, Haviland I, Gubili J (2018). Herbal medicine for depression and anxiety: a systematic review with assessment of potential psycho-oncologic relevance. Phytother Res.

[9] Chiu J, Yau T, Epstein RJ (2009). Complications of traditional Chinese/herbal medicines (TCM)—a guide for perplexed oncologists and other cancer caregivers. Support Care Cancer.

[10] Brett J, Murnion B (2015). Management of benzodiazepine misuse and dependence. Aust Prescr.

[11] Norman IJ, Bergin M, Parry CD, Van Hout MC (2016). Best practices and innovations for managing codeine misuse and dependence. J Pharm Pharm Sci.

[12] Haider M, Shafqat MN (2017). Comparison of duloxetine and SSRI as a treatment option of painful physical symptoms associated with major depressive disorder. Neuropsychiatr Dis Treat.

[13] Foulds J, Steinberg MB, Williams JM, Ziedonis DM (2006). Developments in pharmacotherapy for tobacco dependence: past, present and future. Drug Alcohol Rev.

[14] Ouzir M, Errami M (2016). Etiological theories of addiction: a comprehensive update on neurobiological, genetic and behavioural vulnerability. Pharmacol Biochem Behav.

[15] Leshner AI (1997). Addiction is a brain disease, and it matters. Science.

[16] Wang SZ, Lu YH, Wu M, Chen KJ, Liu Y, Liu LT (2021). Cupping therapy for diseases: an overview of scientific evidence from 2009 to 2019. Chin J Integr Med.

[17] Aboushanab TS, AlSanad S (2018). Cupping therapy: an overview from a modern medicine perspective. J Acupunct Meridian Stud.

[18] Behere RV, Muralidharan K, Benegal V (2009). Complementary and alternative medicine in the treatment of substance use disorders—a review of the evidence. Drug Alcohol Rev.

[19] Lu L, Liu Y, Zhu W, Shi J, Liu Y, Ling W, Kosten TR (2009). Traditional medicine in the treatment of drug addiction. Am J Drug Alcohol Abuse.

[20] Wu J, Hu Y, Zhu Y, Yin P, Litscher G, Xu S (2015). Systematic review of adverse effects: a further step towards modernization of acupuncture in China. Evid Based Complement Alternat Med.

